# Thermodynamic Investigation of an Integrated Solar Combined Cycle with an ORC System

**DOI:** 10.3390/e21040428

**Published:** 2019-04-22

**Authors:** Shucheng Wang, Zhongguang Fu

**Affiliations:** 1 School of Energy Power and Mechanical Engineering, North China Electric Power University, Beijing 102206, China; 2 Section of Thermal Energy, Department of Mechanical Engineering, Technical University of Denmark, Building 403, Nils Koppels Allé, 2800 Kgs. Lyngby, Denmark

**Keywords:** ISCC, CCPP, ORC, thermodynamic analysis, solar energy, waste heat recovery

## Abstract

An integrated solar combined cycle (ISCC) with a low temperature waste heat recovery system is proposed in this paper. The combined system consists of a conventional natural gas combined cycle, organic Rankine cycle and solar fields. The performance of an organic Rankine cycle subsystem as well as the overall proposed ISCC system are analyzed using organic working fluids. Besides, parameters including the pump discharge pressure, exhaust gas temperature, thermal and exergy efficiencies, unit cost of exergy for product and annual CO_2_-savings were considered. Results indicate that Rc318 contributes the highest exhaust gas temperature of 71.2℃, while R113 showed the lowest exhaust gas temperature of 65.89 at 800 W/m^2^, in the proposed ISCC system. The overall plant thermal efficiency increases rapidly with solar radiation, while the exergy efficiency appears to have a downward trend. R227ea had both the largest thermal efficiency of 58.33% and exergy efficiency of 48.09% at 800W/m^2^. In addition, for the organic Rankine cycle, the exergy destructions of the evaporator, turbine and condenser decreased with increasing solar radiation. The evaporator contributed the largest exergy destruction followed by the turbine, condenser and pump. Besides, according to the economic analysis, R227ea had the lowest production cost of 19.3 $/GJ.

## 1. Introduction

World economy growth, energy shortage and environment deterioration are major issues with the development of society [1]. Although, the natural gas combined cycles (NGCCs) produce lower greenhouse gas emissions than that of coal-fired power plants, it is becoming urgent to search for sustainable alternatives to cope with world energy demands as well as the requirement of protecting the environment [2,3]. In this vein, the application of solar energy is perceived to be a promising way of mitigating the negative impacts to the environment and of improving energy security. 

### 1.1. Literaure Review

Solar thermal power plants are facing huge initial investment, as energy storage tends to raise the cost of overall plant. In the past decades, many researchers focused on the integrated solar combined cycle (ISCC) system with theoretical and experimental studies. Integrating solar energy into traditional NGCC can significantly enhance the electrical power generation while reducing the fossil fuel consumption and greenhouse gas emissions [4]. Moreover, ISCC plants do not require the construction of expensive huge heat storage systems. For these reasons, the first ISCC system was built in Italy (the Archimede Project). After that, many other ISCC systems were established in Egypt, Morocco and Florida [5]. In addition, researchers offered valuable technologies and best configurations for the ISCC systems. Aldali et al. [6] analyzed the thermodynamic performance of an ISCC system between the power increasing and fuel-saving modes. Their results showed that the fuel saving mode has a higher benefit/cost ratio for the same solar field area than the boosting model. Montes et al. [7] explored the performance of ISCC using direct steam generation (DSG) technology, revealing that the DSG configurations could reduce the irreversibility of the ISCC, and thus had a better performance.

Previous literature provides significant insights on the advantages of the ISCC technologies, indicating that the ISCC plants have a better performance than the conventional NGCC systems. However, there is still a part of waste heat, which should be utilized to further increase the thermal efficiency. Recently, organic Rankine cycle (ORC) technology has been used for waste heat recovery and the ORC uses an organic working fluid which has a lower boiling point, lower latent heat and small specific volume compared to water. The ORC can select for different components acceptable for various ranges of power output, thus it shows great flexibility in increasing the efficiency of power plants. These advantages enable ORC to make a good use of low-grade energy and has been widely researched.

Esquivel-Patino et al. [8] investigated a 453 MW NGCC integrated with a 260 kW ORC system to generate electricity with the wasted energy, which increased the power generation by 1.65 MW. Song et al. [9] explored the optimization of the supercritical carbon dioxide (S-CO_2_) with a bottoming ORC system to recover the residual heat of the topping S-CO_2_ cycle. The simulation results showed the ORC increased the thermal efficiency by 1.3%, and the thermal performance was improved significantly by raising the ORC evaporation temperature. Moreover, Katulic et al. [10] presented the thermodynamic performance of a bottom cycle with ORC, and they pointed out that it was possible to achieve both higher steam turbine and the overall plant efficiencies by enabling the optimization algorithm to determine the layout of heat exchangers. 

In addition, several novel ORC systems and analysis methods have been proposed by other authors. Shaaban [11] used the ORC to recover the heat of compressed air in the Brayton cycle. The results shown that the R1234ze had a better performance than any other organic fluids. Chowdhury [12] carried out the dynamic performance of ORC using a finite volume method. They concluded the proposed model could capture transient effects of more than 90% of data fitness. Singh and Mishra [13] analyzed the solar driven combined S-CO_2_ and ORC using energy and exergy methods and five organic working fluids were considered. Also, Spayde [14] hourly analyzed a combined solar powered ORC with energy storage. Algieri et al. [15] presented and optimized an ORC with solar collectors and a thermal storage tank. Numerical results showed that the system was capable of generating stable power during the day with a solar fraction of 100%. 

Additionally, ORC has widespread applications in the area of combined cooling heating and power (CCHP) systems to increase the power generation and to improve the thermal efficiency. Fang et al. [16] configured a complementary CCHP-ORC system for a hotel, and showed that the proposed system had a better performance than the conventional CCHP system in the area of fuel consumption, CO_2_ emissions and operation costs. Ebrahimi and Ahookhosh [17] pointed out that the largest exergy destructions were the recuperator and the combustion chamber. Asouri et al. [18] presented an exergo-economic analysis and optimization of a double pressure organic Rankine cycle coupled with a solar collector. Results showed that the system was capable of generating stable power during the day with a solar fraction of 100%. In nights and overcasts, the system can still generate power with the help of the auxiliary heater. 

### 1.2. Motivation

Although, some research have been conducted on ORC systems, few literature reviews of ISCC power plants coupled with the organic Rankine cycle (ISCC-ORC) to recover the waste heat of exhaust gas have been done. In this work, the ORC subsystem is proposed to recovery the low-grade thermal energy in the heat recovery steam generator (HRSG) of an ISCC system, which is uncomplicated to implement in engineering and minimizes its impact on the original system. The ISCC is consisted of a conventional NGCC system and a solar field. After a long period of operation in many NGCC power plants, the temperature of exhaust gas will rise. How to further recover this part of energy and improve the thermal efficiency conveniently is an urgent issue. Moreover, the changing solar radiation intensity (SRI) impacts on the performance of the ISCC-ORC system. Therefore, it is an important issue to better understand the performance of the ISCC-ORC system. 

This paper focuses its attention on the thermodynamic and economic performance of the ORC as well as the proposed ISCC-ORC plant. The bottoming ORC has been proposed to recover the low-grade energy (exhaust gas of the ISCC). Additionally, in order to enhance the system performance and reveal the irreversibility in each component of the ORC subsystem, the exergy destruction of ORC components, energy and exergy efficiencies have been assessed and compared under specific SRI. The results can provide significant theoretical guidance for the renovation of gas-fired power plants.

## 2. System Description 

**Figure 1:**
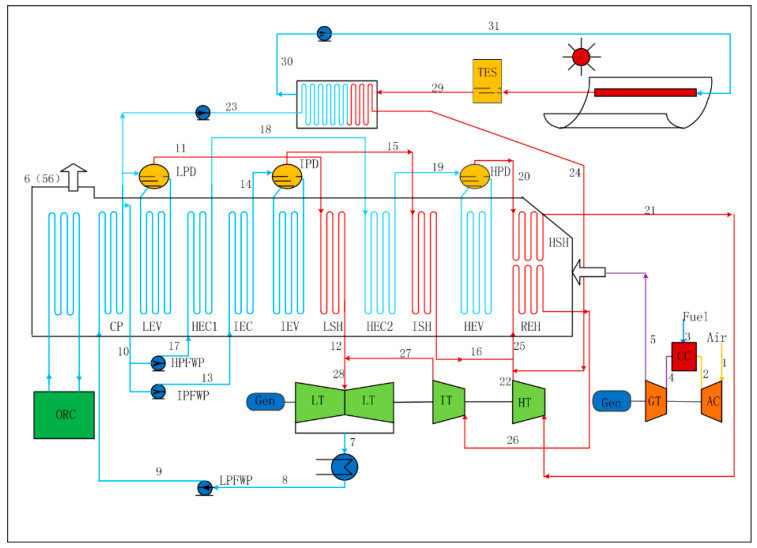
The schematic of an integrated solar combined cycle with an organic Rankine cycle.

The proposed ISCC-ORC plant consists of a traditional NGCC (SGT5-4000F), a solar field and an ORC subsystem, as depicted in Figure 1. The exhaust gas temperature of the NGCC is 91 ℃ (designed value), however, after a long period of operation, the efficiency of the system decreases, and the exhaust temperature rises up to 110 ℃ under full-load conditions. Therefore, an ORC subsystem is proposed to further use this excessive energy and reduce the temperature of exhaust gas. Besides, the ORC subsystem can be installed at the end of the heat recovery steam generator (HRSG), minimizing its impact on the original system.

### 2.1. Integrated Solar Combined Cycle System

The ISCC system consists of a conventional NGCC which includes a Siemens V94.3A, a steam turbine, a three-pressure HRSG with reheat and a solar field. The fuel burns with the compressed air in the combustion chamber (CC), and the exhaust gas of the CC with high temperature and high pressure expands in the gas turbine (GT), which then drives the air compressor (AC) and the generator. The exhaust gas of the GT is introduced to the HRSG to generate steam with different pressure levels, i.e., high-pressure steam, intermediate-pressure steam and low-pressure steam. Meanwhile, a solar field with thermal energy storage (TES) is used to heat part of the feed water. Then the heated water from solar field mix with the exhaust steam of high-pressure turbine and the intermediate-pressure steam. After that, the mixed steam is introduced to the reheater before injection into the intermediate-pressure turbine. Herein, it should be noted that the pressure between the solar field superheated steam (2.92 MPa, 358.8 °C) and the exhaust steam of high-pressure steam turbine (2.92 MPa, 361.5 °C) are the same, and the temperatures are similar to minimize the energy losses.

### 2.2. The Organic Rankine Cycle Subsystem

**Figure 2:**
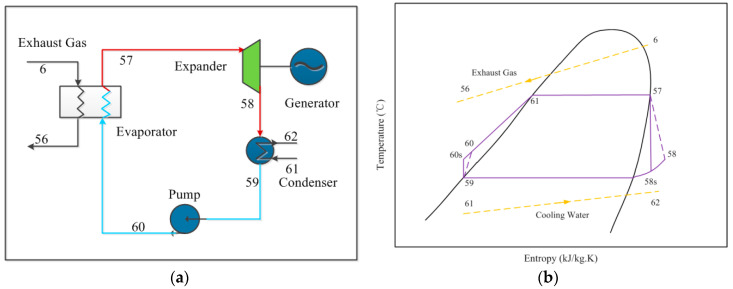
(**a**) Schematic of an ORC system. (**b**) Temperature-Entropy diagram of an ORC system.

The ORC is a kind of Rankine cycle using organic fluids as the working fluids, which have a lower boiling point. The ORC subsystem consists of an evaporator, a condenser, a pump and a turbine, as shown in Figure 2a. The working process starts from the compressed fluid heated by the exhaust gas from the HRSG, then the working fluid expands in the expander and drives the generator to produce power. Afterwards, the expansive steam is liquefied by the condenser. The thermodynamic processes for a Temperature-Entropy diagram of ORC is depicted in Figure 2b. The working process can be divided into four parts: Isentropic compression (59–60). Isobaric endothermic (60–61–57), Isentropic expansion (57–58s) and Isobaric exothermic (58s–59). Besides, the exhaust temperature of ISCC reduces from point 6 to point 56, and the cooling water of condenser increases from point 61 to point 62. Besides, different organic working fluids including R113, R245ca, RC318 and R227ea are testified to demonstrate their influence on the ORC system performance. 

## 3. Modeling Methodology

### 3.1. ISCC Subsystem Modeling

The ISCC subsystem consists of a conventional NGCC and a solar field, and the ISCC subsystem modeling was referred our previous work [19]. For the solar field subsystem, the received solar energy can be calculated by:

(1) $$Q_{s}=A\cdot N\cdot DNI$$

where A represents the effective area of single solar collector, N represents the total number of collectors and *DNI* stands for the direct normal irradiance.

For the solar field, the absorbed solar energy $Q_{a}$ is calculated as:

(2) $$Q_{a}=\eta_{\mathrm{opt}}\cdot Q_{s}$$

where the $\eta_{\mathrm{opt}}$ is the efficiency of the collector optical and it can be further expressed as:

(3) $$\eta_{\mathrm{opt}}=\eta_{\mathrm{int}}\cdot \eta_{\mathrm{ref}}\cdot \eta_{\mathrm{tra}}\cdot \eta_{\mathrm{abs}}\cdot \eta_{\mathrm{clc}}\cdot \eta_{\phi }\cdot K$$

where $\eta_{\mathrm{int}}$, $\eta_{\mathrm{ref}}$, $\eta_{\mathrm{tra}}$, $\eta_{\mathrm{abs}}$, $\eta_{\mathrm{clc}}$, $\eta_{\phi }$ and *K* are intercept factor, mirror reflectivity, glass transmissivity, solar absorptivity, solar absorptivity, clean factor, mirror utilization rate and incidence angle modifier, respectively.

The incident angle modifier for the examined collectors, LS-2, is given according to the following equation, as a function of the incident angle (θ) in degrees [7]:

(4) $$K(\theta )=\cos(\theta )-5.25097\cdot 10^{-4}\cdot \theta -2.859621\cdot 10^{-5}\cdot \theta^{2}$$

where the incident angle (θ) is calculated by Equation (5) [7]:

(5) $$\cos(\theta )=\sqrt{\cos^{2}(\theta_{z})+\cos^{2}(\delta )\cdot \sin^{2}(\omega )}$$

where δ is the solar declination angle and it is calculated according to Equation (6), with DD to be the number of the day in the year (for instance: DD = 1 for 1 January) [20]:

(6) $$\delta =23.45\cdot \sin(2\pi \frac{284+\mathrm{DD}}{365})$$

The solar angle (ω) is calculated as the following equation, and the solar time ($t_{h}$) is considered [20]:

(7) $$\omega =15\cdot (t_{h}-12)$$

Additionally, the energy efficiency of the ISCC subsystem is defined as the ratio of net output power to the total energy input the ISCC subsystem:

(8) $$\eta_{\mathrm{ISCCS}}=\frac{W_{\mathrm{net}}}{m_{f}\cdot LHV_{f}+Q_{s}}$$

where $m_{f}$ is the natural gas consumption rate, kg/s, $LHV_{f}$ is the lower heating value of natural gas, and $Q_{a}$ stands for the absorbed solar energy of solar field, respectively.

The exergy analysis method differs from the energy analysis method in a way that it considers the second law of the thermodynamics. It is based on both the first and second law of the thermodynamics. The exergy analysis method can contribute a significant assessment, both quantitatively and qualitatively, of elements existing in the energy conversion process.

The exergy of solar radiation absorbed by the receiver tube can be calculated by the Petela’s formula which is expressed as [21]:

(9) $$Ex_{a}=Q_{s}\cdot \psi$$

where for the ideal process, $Q_{s}$ is the received solar energy. The ψ stands for the maximum work obtained from the solar radiation and it can be further written by [21]:

(10) $$\psi =1-\frac{4}{3}\frac{T_{0}}{T_{s}}+\frac{1}{3}{\left(\frac{T_{0}}{T_{s}}\right)}^{4}$$

where $T_{0}$ and $T_{s}$ are the ambition temperature and the equivalent temperature of the sun as a black body (~5770 K), respectively.

The exergy can also be explained as the portion of energy that can be absolutely converted to another form of energy. In addition, the value of exergy consists of four portions, the kinetic and potential exergy, and the physical and chemical exergy. It should be noticed that the changing of velocity and elevation can be neglected. Since, the overall system does not have nozzles and diffusers, which cause tremendous changes in velocity. Therefore, the exergy balance equation can be expressed as:

(11) $$\dot{E}x=\dot{E}x_{\mathrm{ph}}+\dot{E}x_{\mathrm{ch}}$$

where $\dot{E}x_{\mathrm{ph}}$, $\dot{E}x_{\mathrm{ch}}$ are the physical exergy and the chemical exergy which can be further obtained by the following equation [22]:

(12) $$\dot{E}x_{\mathrm{ph}}=\dot{m}\cdot [(h-h_{0}-T_{0}(s-s_{0})]$$

(13) $$\dot{E}x_{\mathrm{ch}}=\dot{m}\cdot [\sum_{i=1}^{n}x_{i}ex_{i}+RT_{0}\sum_{i=1}^{n}x_{i}\ln x_{i}]$$

where T is the absolute temperature (K) and (_0_) refers to the ambient conditions; $\dot{m}$ is the mass flow rate of fluid; $ex_{i}$ is the chemical exergy of *i*-th constituent; and R is the molar gas constant.

However, using the above equation it is difficult to estimate the chemical exergy. Consequently, the following equation is presented to simplify the calculation process:

(14) $$\dot{E}x_{f}=\xi \cdot LHV_{f}$$

where ξ is the ratio coefficient of fossil fuel chemical exergy to the lower heating value (the ratio coefficient for natural gas is 1.06).

### 3.2. ORC Subsystem Modeling

The ORC subsystem has the same principle as the traditional Rankine cycle, and the evaporator, expander, condenser and the pump are the main components. The working processes of ORC subsystem can be divided as: (1) Isentropic compression, (2) Isobaric endothermic, (3) Isentropic expansion and (4) Isobaric exothermic.

For the isentropic compression, the power consumed by the pump $W_{p}$ can be defined as [16]:

(15) $$W_{p}=m_{\mathrm{orc}}(h_{60}-h_{59})=\frac{m_{\mathrm{orc}}(h_{60s}-h_{59})}{\eta_{p}}$$

while the $m_{\mathrm{orc}}$ is the mass flow rate of the organic fluid, $h_{59}$ is the enthalpy of the organic working fluid at the inlet of the pump, $h_{60}$ represents the enthalpy of the organic working fluid at the outlet of the pump, $h_{60s}$ stands for the enthalpy at the outlet of pump under isentropic conditions.

The working organic fluid recovers the heat from the exhaust gas of the ISCC. $Q_{\mathrm{ro}}$ stands for the used heat from the exhaust gas (evaporator), and the absorbed heat $Q_{\mathrm{ep}}$ is expressed as [16]:

(16) $$Q_{\mathrm{ep}}=Q_{\mathrm{ro}}\cdot \xi =m_{\mathrm{orc}}\left(h_{57}-h_{60}\right)$$

where, ξ is the efficiency of the evaporator, and $h_{57}$ represents the enthalpy of the organic working fluid at the outlet of the evaporator.

For the condenser, the heat taken away from condensate water can be calculated by the following equation [19]:

(17) $$Q_{\mathrm{cd}}=m_{\mathrm{orc}}(h_{58}-h_{59})$$

where $h_{58}$ represents the enthalpy of organic working fluid at the outlet of the expander.

The power output of the expander $W_{t}$ can be written as [16]:

(18) $$W_{t}=m_{\mathrm{orc}}(h_{57}-h_{58})=m_{\mathrm{orc}}(h_{57}-h_{58s})$$

where $h_{58s}$ represents the enthalpy of organic working fluid at the outlet of the expander under isentropic conditions.

Additionally, the overall ORC subsystem thermal efficiency is given by [16]:

(19) $$\eta_{\mathrm{orc}}=\frac{W_{t}-W_{p}}{Q_{\mathrm{ep}}}=\frac{(h_{57}-h_{58s})\eta_{t}-(h_{60s}-h_{59})\eta_{p}^{-1}}{h_{57}-h_{60}}$$

The power output of the ORC subsystem can be calculated as [16]:

(20) $$E_{\mathrm{orc}}=Q_{\mathrm{ro}}\eta_{\mathrm{orc}}\eta_{\mathrm{gen}}$$

where $\eta_{\mathrm{gen}}$ is the efficiency of the power generator. 

The exergy destruction in the components of proposed system is considered and the exergy balance equation for *k*-th component is defined as [22]:

(21) $$\dot{E}x_{D,k}=\dot{E}x_{F,k}-\dot{E}x_{p,k}$$

where, for *k*-th component, the $\dot{E}x_{F,k}$ stands for “fuel exergy” which is the energy consumed in the energy conversion process, while the $\dot{E}x_{p,k}$ stands for “product exergy”.

For better understanding the exergy destruction, the exergy efficiency and the exergy destruction rate for the *k*-th component are defined as follows [22]:

(22) $$\eta_{e}=\dot{E}x_{p,k}/\dot{E}x_{F,k}$$

(23) $$y_{D,k}={\dot{E}}_{D,k}/{\dot{E}}_{F,k}$$

## 4. Results and Discussion

### 4.1. ISCC Model

The model of ISCC consists of a conventional NGCC (SGT5-4000F of Siemens) and a solar field (parabolic trough collector, PTC) as depicted in Figure 1. Additionally, the proposed ISCC system was established in the Ebsilon *Professional* software. Herein, the main thermodynamic parameters of proposed system have been selected to verify the simulation results as shown in Table 1 and Table 2. The validation of the NGCC model has been possible through the comparison of current results with the design values of Siemens from the China Energy Investment Corporation. It is shown that the simulation values of current model shows good agreement with the design values of Siemens. 

In this study, the LS-2 solar collector (PTC) was selected to track the sun from east to west. The main thermodynamic parameters of the PTC are shown in Table 2. Particularly, the PTCs were uniformed on North-South lines. The simulation location was Shanghai, China (31.2° N, 121.4° E) with an ambient temperature of 20 °C and wind speed of 2.2 m/s. Additionally, the length and the width of the PTC are 150 m and 5.76 m, respectively. The inlet and outlet temperature of the solar field is 149.5 °C and 358.5 °C, the heat losses are 20 W/m^2^. The thermal energy storage capacity is 40 L/kWh. Herein, the outlet temperature of the superheated steam from solar field is constant. While, the mass flow rate of the steam can vary with the SRI. 

**Figure 3:**
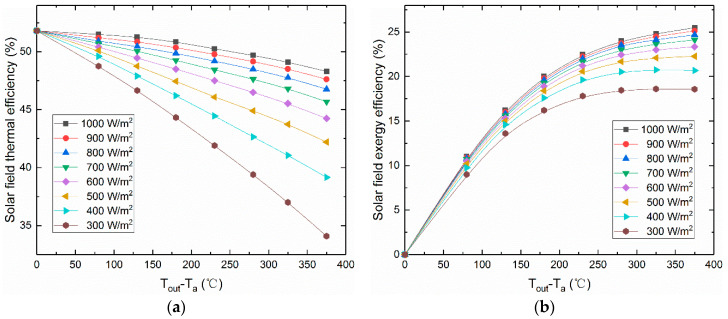
Thermal and exergy efficiencies of the solar field for various operating conditions. (**a**) The thermal efficiency of the solar field under various operation conditions. (**b**) The exergy efficiency of the solar field under various operation conditions.

The thermal and exergy efficiencies under different SRIs are depicted in Figure 3. It is clear that the thermal efficiency of the solar field is high enough in upper SRI and it decreases rapidly under low SRI. That is to say, the smaller the temperature difference between the feed water and the ambient, the smaller the heat loss in the solar collectors is. Therefore, for the solar collectors, a lower temperature difference point shows a higher thermal efficiency. In addition, the solar field exergy efficiency shows satisfactory values only at high-temperature levels, while the exergy efficiency keeps values close to zero in low outlet temperature levels. A higher outlet temperature of the solar field can produce more useful work.

Besides, the thermal efficiency is equal to 51.8% and the exergy efficiency is equal to zero at the point of T_out_ − T_a_ = 0. The reason is that the inlet and outlet temperature levels of the solar field are close to the ambient temperature (T_a_), this point has the least energy loss and there is no useful work significantly produced in this point. 

**Figure 4:**
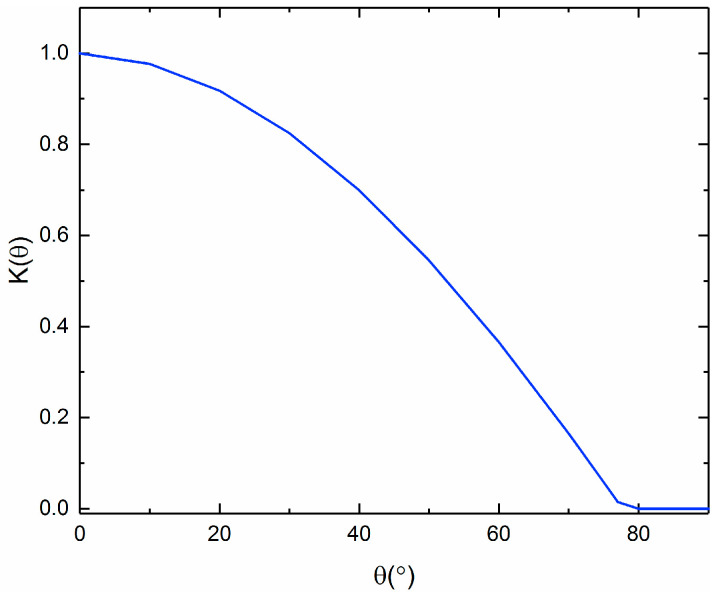
Incident angle modifier of the collector for various incident angles.

Figure 4 depicts the incident angle modifier for various incident angles, according to Equation (4). It is obvious that the incident angle modifier is equal to 1 at the point of θ = 0, and it decreases to zero after θ = 80°.

### 4.2. ORC Model 

Herein, the ORC subsystem was used for the recovery of the waste heat from the exhaust gas of a HRSG. The ORC model was chosen from the Ebsilon library. The proposed ORC subsystem can further reduce the temperature, due to the soot and little sulfur products from natural gas combustion. The thermal parameters of the ORC system are listed in Table 3. The efficiencies of turbine and pump are from the literature of Singh [13]. 

**Figure 5:**
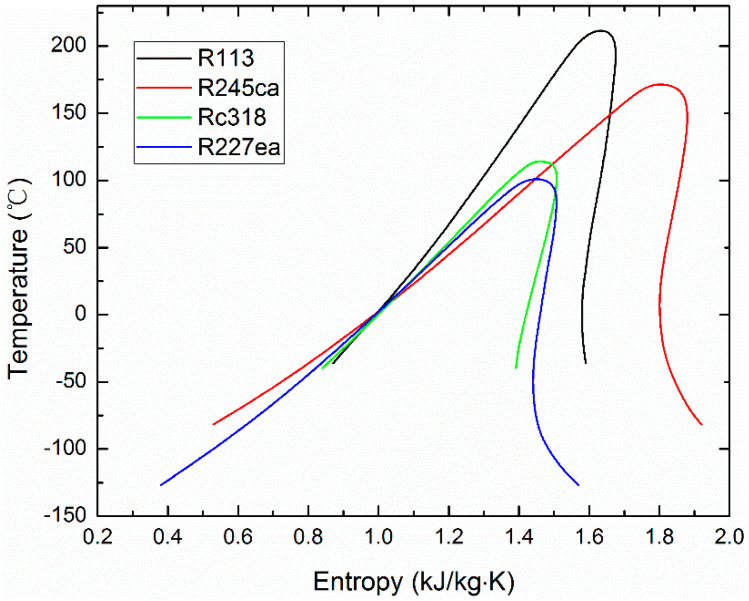
Temperature-Entropy diagram of the considered organic working fluids.

To better understand the effects of different organic working fluid on the performance of the proposed ISCC-ORC system, organic fluids including R113, R245ca, Rc318 and R227ea are considered as the research objects according to the properties and parameters of the working fluid. The thermodynamic parameters of selected organic working fluid are shown in Table 4. Additionally, the T-S diagram of the considered organic working fluids are shown in Figure 5.

### 4.3. Energy and Exergy Analysis Results of the ISCC-ORC System

In this section, the model of ISCC-ORC is used to analyze the thermodynamic performance via the energy and exergy analysis methods under different SRI values. However, the thermodynamic parameters of an overall plant will be changed with the variation of SRI. Therefore, the parameters of the exhaust gas temperature, exergy destruction, thermal efficiency and the exergy efficiency of overall plant should be further improved. 

**Figure 6:**
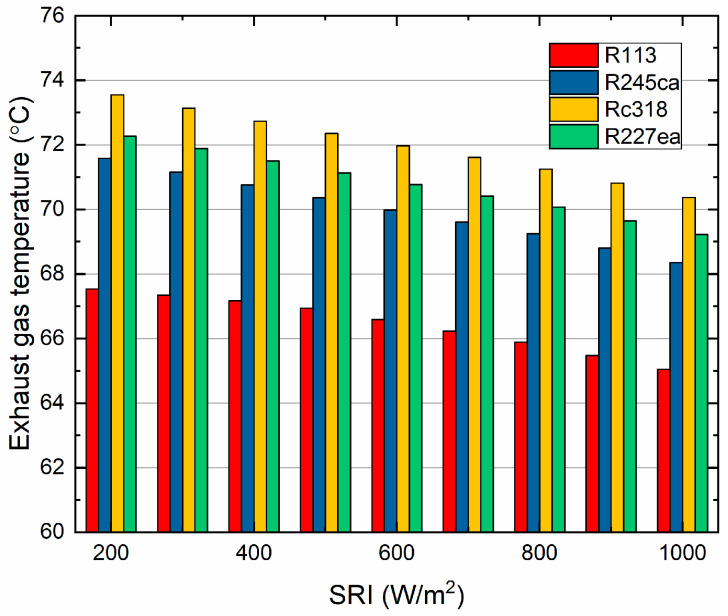
The exhaust gas temperature of the ISCC-ORC system varies with the SRI.

The exhaust gas temperature of the proposed ISCC-ORC system varied with the SRI is shown in Figure 6. It can be observed that the exhaust gas temperature of the four working fluids showed downward trends with increasing SRI. As such, the exhaust gas temperature of Rc318 decreases from 73.5 °C to 70.3 °C when the SRI increased from 200 W/m^2^ to 1000 W/m^2^. And the Rc318 always contributed the highest exhaust gas temperature among other working fluids. Meanwhile, the R113 showed the lowest temperature from 67.5 °C to 65.0 °C. For the reason that the R113 has the highest boiling point (as shown in Table 4) and it needs more energy to reach this boiling point. Therefore, R113 absorbed more heat from exhaust gas than the other working fluids, causing the lowest exhaust gas temperature. However, it can be noticed that the Rc318 ha a higher boiling point, more than that of R227ea, while the exhaust gas temperature of the Rc318 was higher than that of the R227ea. The enthalpy of RC318 increased by 68 kJ/kg, which was higher than that of RC318 (64 kJ/kg).

**Figure 7:**
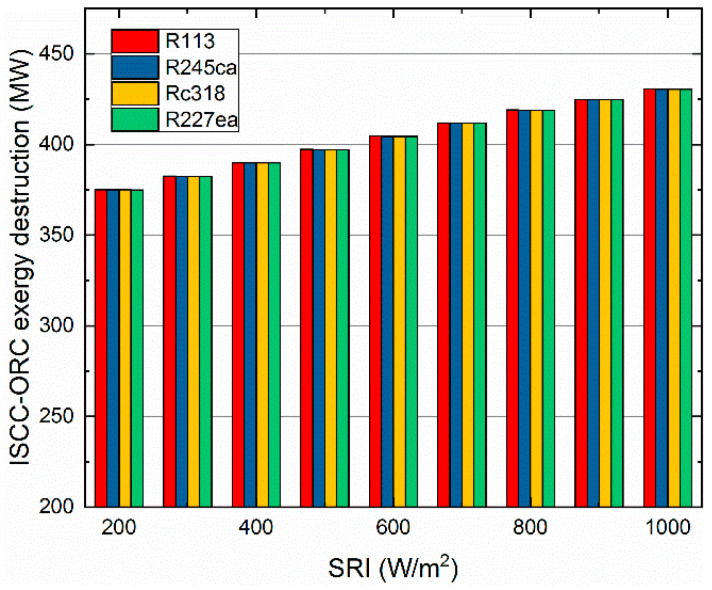
The exergy destruction of the ISCC-ORC system varies with the SRI.

The exergy destruction of the ISCC-ORC system varies with the SRI is demonstrated in Figure 7. It can be seen that the exergy destruction of the four working fluids was similar under specific SRI. As such, the exergy destruction of R113, R245ca, Rc318 and the R227ea were 419.07 MW, 419.02 MW, 418.98 MW and 418.95 MW at 800 W/m^2^. While, the overall plant exergy destruction increased with the SRI, for the solar field had large energy losses both in the oil-water exchanger and the heat transfer process.

**Figure 8:**
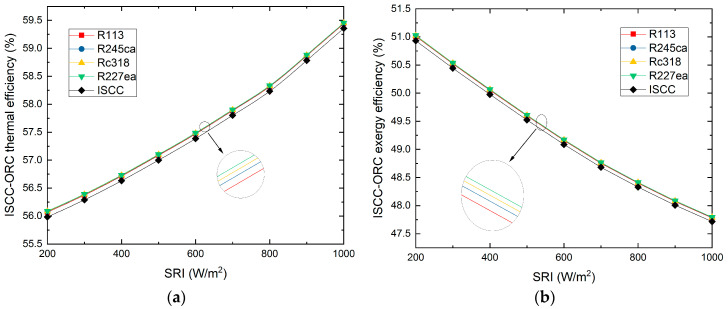
The ISCC-ORC system thermal and exergy efficiencies vary with the SRI. (**a**) The thermal efficiency of the ISCC-ORC system varies with the SRI. (**b**) The exergy efficiency of the ISCC-ORC system varies with the SRI.

The thermal efficiency of proposed ISCC-ORC system varies with the SRI is exhibited in Figure 8a. It reveals that the thermal efficiency of overall plant increases rapidly with the SRI and the working fluid of R227ea appears the largest thermal efficiency (58.33%, 800W/m^2^) than that of others, for the solar field can acquire more energy with the SRI increasing, which increases the solar field efficiency. Besides, it can be also observed that the thermal efficiency of R113, R245ca, Rc318 and R227ea are 58.315%, 58.323%, 58.329% and 58.334% under the SRI value of 800W/m^2^. 

Furthermore, the exergy efficiency of the proposed ISCC-ORC system varies with the SRI, which is exhibited in Figure 8b. It can be seen that the overall plant exergy efficiency shows a downward trend with the SRI increasing, and R227ea contributes the largest exergy efficiency of 48.09% at 800 W/m^2^. Besides, it shows that both the thermal and exergy efficiencies of the proposed ISCC-ORC system are always higher than the compared ISCC system.

### 4.4. Energy and Exergy Results of the ORC Subsystem

In this section, the thermodynamic performance of the proposed ORC subsystem is investigated via the energy and exergy analysis methods under different SRI values and different pump discharge pressures. Particularly, to better understand the performance of the ORC, the power generation, thermal efficiency, exergy efficiency and exergy destruction are investigated. 

**Figure 9:**
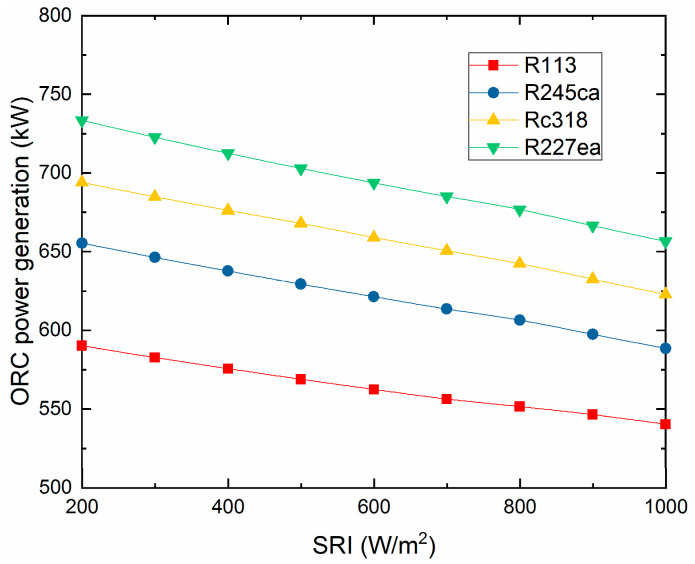
The ORC subsystem power generation varies with the SRI.

The power output of the ORC subsystem varies with the SRI, which is demonstrated in Figure 9. It can be seen that the ORC power generation decreases with the SRI increasing, and the R227ea contributes a highest power generation of 676.7 kW at 800 W/m^2^. The reason for this downward trend is that the natural gas consumption reduces with the SRI increasing, which decreases the exhaust gas temperature of the ISCC.

**Figure 10:**
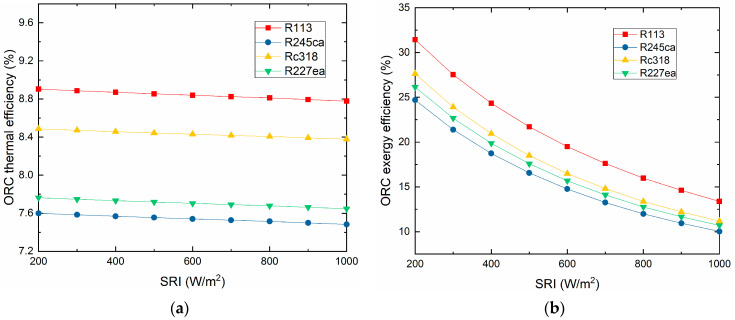
The ORC subsystem thermal and exergy efficiencies vary with the SRI. (**a**) The ORC subsystem thermal efficiency varies with the SRI. (**b**) The ORC subsystem exergy efficiency varies with the SRI.

The thermal efficiency and exergy efficiency of the ORC subsystem vary with the SRI, which is exhibited in Figure 10. It can be seen that the exergy efficiency decreases with the SRI increasing. However, the thermal efficiency of the ORC changes slightly. Additionally, the R113 shows the maximum thermal efficiency (8.81%) and maximum exergy efficiency (15.98%) at 800 W/m^2^. Meanwhile, the R245ca appears both the minimum thermal efficiency (7.51%) and minimum exergy efficiency (11.99%), respectively.

**Figure 11:**
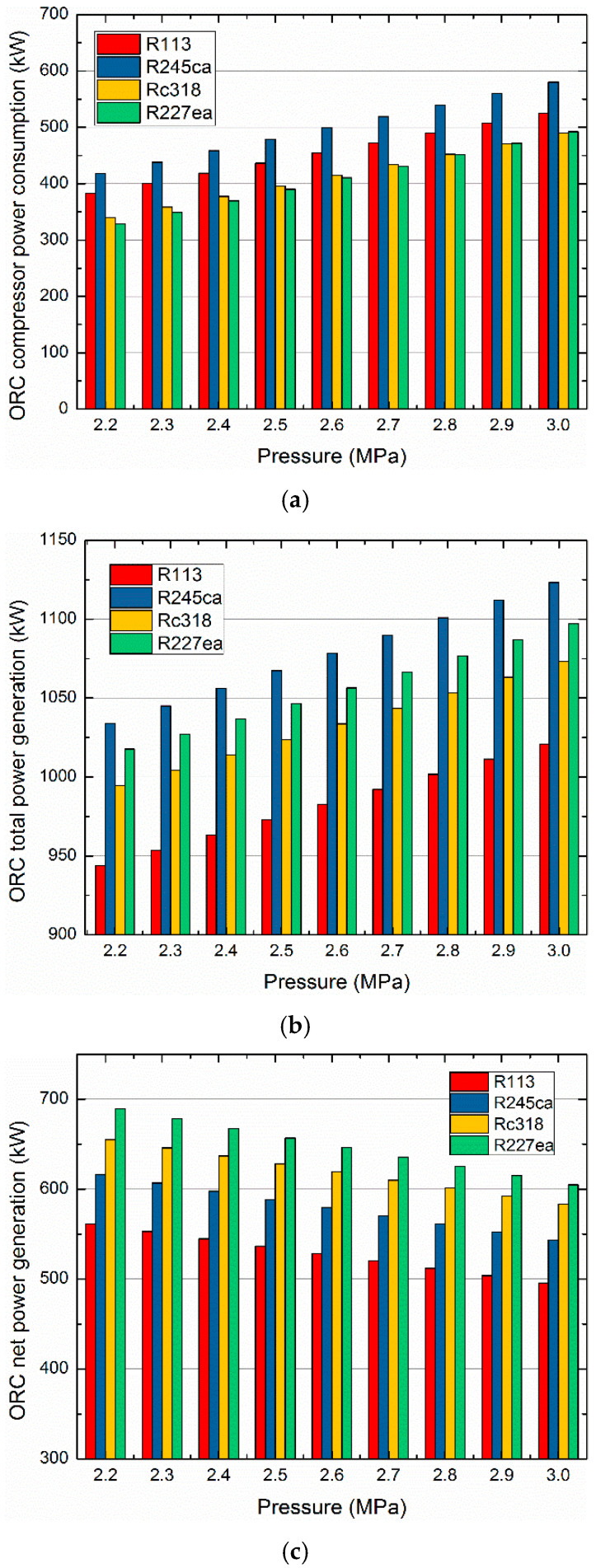
Compressor power consumption, total power generation and net power generation of the ORC vary with the pump discharge pressure. (**a**) ORC compressor power consumption varies with the pump discharge pressure. (**b**) ORC total power generation varies with the pump discharge pressure. (**c**) ORC net power generation varies the pump discharge pressure.

The compressor power consumption, total power generation and net power generation of the ORC subsystem vary with the pump discharge pressure are demonstrated in Figure 11. It is revealed that the compressor power consumption and the total power generation of ORC increase with the raising discharge pressure of pump. While, the ORC net power generation shows an opposite trend. It can be observed that R245ca contributes the largest total power generation and consumes the most energy. However, R227ea has the largest net power generation, since R227ea has the lowest compressor consumption. It should be noticed that the energy consumed by the compressor plays an important role in the net power output of the ORC subsystem.

**Figure 12:**
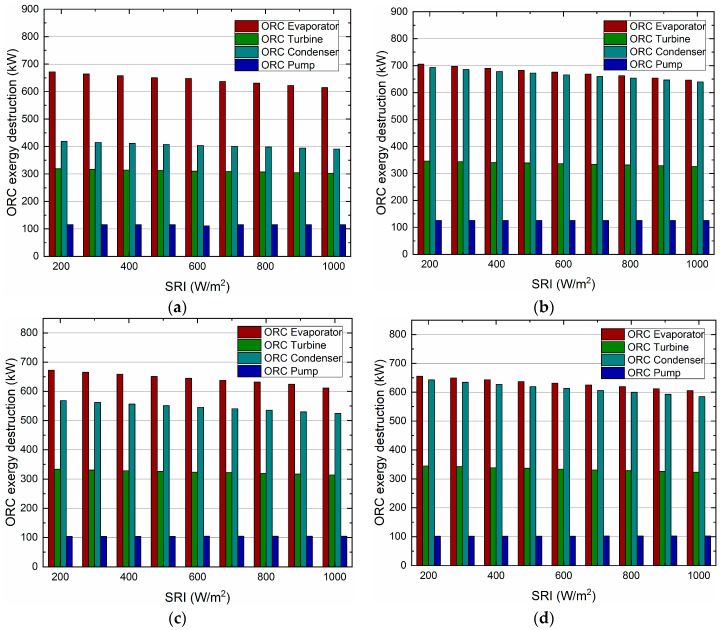
The exergy destruction of main ORC components varies with the SRI for different organic working fluids. (**a**) Organic working fluid of R113. (**b**) Organic working fluid of R245ca. (**c**) Organic working fluid of Rc318. (**d**) Organic working fluid of R227ea.

The exergy destruction of the main ORC components varies with the SRI for different organic working fluids, and is illustrated in Figure 12. It can be observed that the exergy destruction of the evaporator, turbine and condenser decrease with the increasing of the SRI, while the exergy destruction of pump changes slightly. In addition, the evaporator contributes the largest exergy destruction among all the selected organic working fluids followed by the turbine, the condenser and the pump. The reason for the temperature difference between the exhaust gas and the organic working fluid is the large amount of energy loss in the evaporator. The larger the temperature difference, the greater the exergy destruction is. Therefore, the evaporator should be further investigated to improve its thermal and exergy performance. On the contrary, the pump presents the lowest exergy destruction, since it has both effective isentropic and mechanical efficiencies.

Additionally, it can be also observed that R113 (a) contributes the lowest exergy destruction of 307.4kW in a turbine at 800 W/m^2^. For the reason that R113 has better performance in recovering the waste heat below 200 °C, thus decreasing the exergy destruction of the turbine. Meanwhile, the R227ea (d) provides the lowest exergy destruction in the evaporator (619.6 kW) and pump (102.2 kW).

### 4.5. Economic Analysis

The unit cost of exergy for the product is considered to determine the economic performance of the ORC subsystem and it can be illustrated as [27]:

(24) $$c_{\mathrm{product}}=\frac{{\dot{C}}_{\mathrm{product}}}{{\dot{W}}_{\mathrm{net}}}$$

where ${\dot{C}}_{\mathrm{product}}$ is the product cost rate and it can be further calculated by the following equation:

(25) $${\dot{C}}_{\mathrm{product}}={\dot{C}}_{\mathrm{fuel}}+\sum \left({\dot{Z}}_{\mathrm{CI}}+{\dot{Z}}_{\mathrm{OM}}\right)$$

where ${\dot{C}}_{\mathrm{fuel}}$ is the fuel cost rate, ${\dot{Z}}_{\mathrm{CI}}$ and ${\dot{Z}}_{\mathrm{OM}}$ are the sum of the capital investment cost rates and the operating and maintenance cost rates, respectively. It can be expressed as:

(26) $${\dot{Z}}_{\mathrm{CI}}+{\dot{Z}}_{\mathrm{OM}}=\frac{Z_{\mathrm{CI}}\cdot \varphi \cdot CRF}{3600N}$$

where φ is maintenance factor, *N* is the operating hours, $Z_{\mathrm{CI}}$ stands for the capital investment cost of main ORC components, and the cost functions are listed in Table 5. According to Liu’s work [28], the heat exchanger area of the evaporator (${\dot{A}}_{\mathrm{Evap}}$) is assumed to be 84.2 m^2^ and the heat exchanger area of the condenser (${\dot{A}}_{\mathrm{Cond}}$) is estimated as 376.0 m^2^. The *CRF* is the capital recovery factor, which can be defined by the Equation (27):

(27) $$CRF=\frac{i{\left(1+i\right)}^{n}}{{\left(1+i\right)}^{n}-1}$$

where the *i* is the annual interest rate, *n* is the system serve life. In addition, the main parameters selected in economic analysis are shown in Table 6.

**Figure 13:**
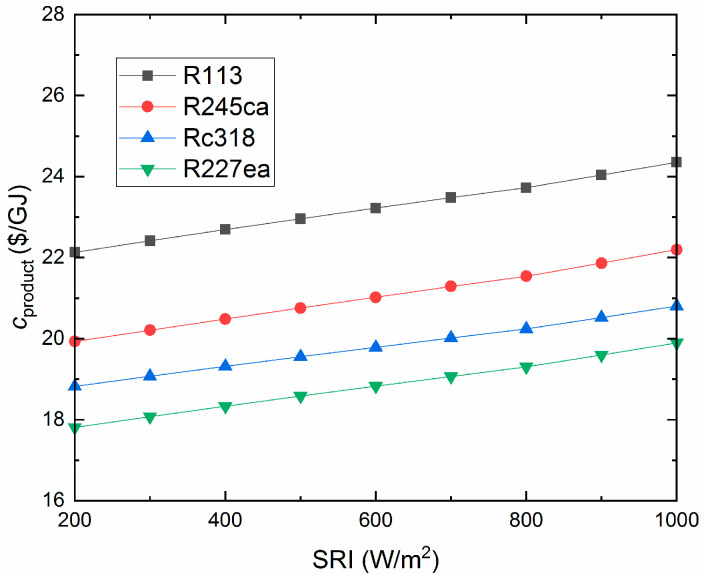
The unit cost of exergy for product System.

**Figure 14:**
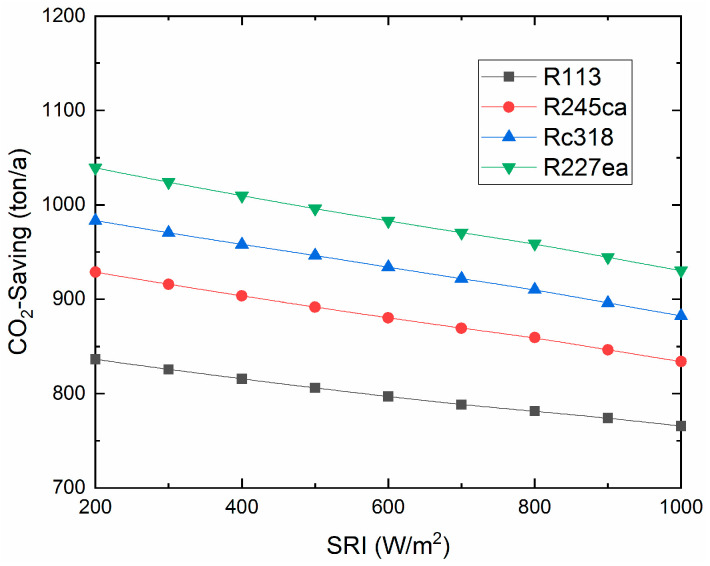
CO_2_-saving for the ISCC-ORC varies with the SRI.

The total production cost varies with the SRI, as shown in Figure 13. It can be seen that the total production cost increases with the SRI increasing. Simultaneously, it is noteworthy that R227ea has the lowest production cost (19.3 $/GJ, 800W/m^2^). The CO_2_-saving of the proposed system is shown in Figure 14. R227ea can save the largest CO_2_ (958.7ton/a, 800W/m^2^), for the reason that R227ea produces the most electricity among the four working fluids. Besides, the annual CO_2_-saving decreases with the SRI, for the ISCC-ORC system would consume more natural gas and produce more CO_2_ under low SRI. However, as a kind of clean energy, the more solar energy input to the proposed system, the higher efficiency it will be.

**Figure 15:**
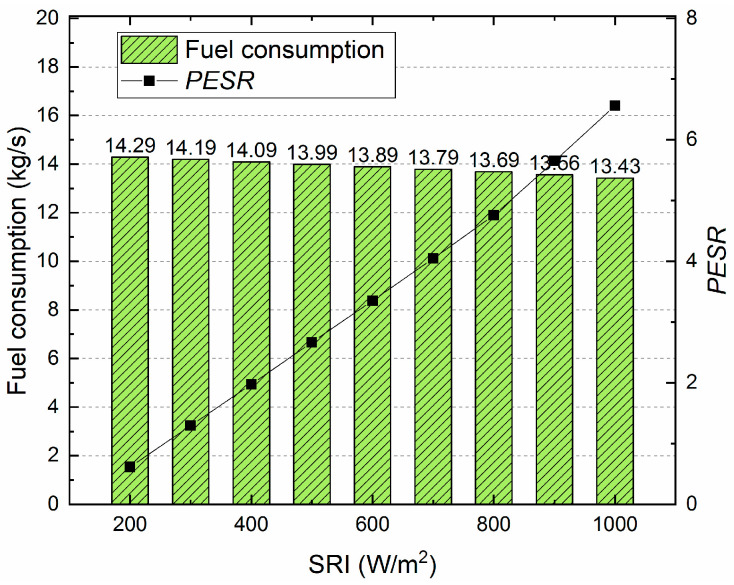
The fuel consumption and PESR under different SRI.

Herein, R227ea is selected as the working fluid in the proposed system due to its higher performance shown in the above analysis. And the results of fuel consumption and primary energy saving ratio (PESR) under different SRI is shown in Figure 15. It can be seen that the fuel consumption drops from 14.29 kg/s (200 W/m^2^) to 13.43 kg/s (1000 W/m^2^), and the PESR increases from 0.61% (200 W/m^2^) to 6.56% (1000 W/m^2^).

## 5. Conclusions

In this paper, an ISCC-ORC system consisting of conventional NGCC, solar field and ORC subsystem is proposed. The variation performance of ORC subsystem and overall proposed ISCC-ORC system are analyzed via the thermodynamic and economic analysis methods under specific SRIs. In addition, organic working fluids, pump discharge pressures, exhaust gas temperature, thermal and exergy efficiencies, unit cost of exergy for product and annual CO_2_-saving are considered. 

Additionally, in the application of any technology, the designers must consider the issues of protecting the environment and avoiding the use of harmful materials. In this research, we investigated the performance between organic fluids, which is not enough. In future work, we will further analyze of all the common organic working fluids which will be analyzed for providing suggestions for applications. From the obtained results, the conclusions of this research are as follows:

(a) For the overall plant, the exhaust gas temperature of the four working fluids showed downward trends with increasing SRI. Rc318 and R113 contributed the highest and lowest exhaust gas temperature, respectively.
(b) The overall plant thermal efficiency increased rapidly with the SRI, while the exergy efficiency showed the opposite trend. The R227ea appeared to have the largest thermal efficiency of 58.33% and the highest exergy efficiency of 48.09%, at 800 W/m^2^. Moreover, the proposed ISCC-ORC system always had a better performance than the ISCC.
(c) For the ORC subsystem, the compressor power consumption and total power generation increased with the pump discharge pressure. While, the ORC net power generation showed an opposite trend. Additionally, the energy consumed by the compressor had a dominant role in the net power output of the ORC subsystem.
(d) Both the thermal efficiency and exergy efficiency of the ORC decreased with the increasing of the SRI. Additionally, R113 showed the maximum thermal efficiency (8.81%) and exergy efficiency of (15.98%) at 800 W/m^2^. Meanwhile, R245ca appeared to have the minimum thermal efficiency (7.51%) and exergy efficiency (11.99%), respectively.
(e) The exergy destruction of the evaporator, turbine and condenser decreased with the increasing of the SRI. In addition, the evaporator contributed the largest exergy destruction among the selected organic working fluids followed by the turbine, condenser and pump. Besides, among all considered refrigerants, R245ca corresponded to the largest exergy destruction of the ORC system.
(f) The unit cost of exergy for the product and the CO_2_-saving are decreased with the SRI. R227ea has the lowest production cost (19.3$/GJ, 800W/m^2^) and R227ea can save the largest amount of CO_2_ (958.7ton/a, 800W/m^2^).

## Figures and Tables

**Table 1:** Main thermodynamic parameters of SGT5-4000F.

Parameters	Siemens	Simulation	Units
Capacity	390	390	MW
Main steam Reheated steam Low-pressure steam	12.5/566/72.6 2.99/551/85.6 0.45/239/12.3	12.6/567/73.8 2.91/551/86.7 0.46/239.9/12.9	MPa/°C/kg·s^−1^ MPa/°C/kg·s^−1^ MPa/°C/kg·s^−1^
Gas turbine exhaust Ambient temperature	590/643 20	590.6/646 20	°C/kg·s^−1^ °C
Exhaust gas temperature	91	90.9	°C

**Table 2:** Main parameters of parabolic trough collectors.

Parameters	Values	Ref.
Width	5.76 m	[13]
Length	150 m	-
Number of collectors	280	-
Temperature of feed water	149.5 °C	
Temperature of superheated steam	358.5 °C	-
Intercept factor $\eta_{\mathrm{int}}$	0.92	[7,23]
Mirror reflectivity $\eta_{\mathrm{ref}}$	0.92	[7,23]
Glass transmissivity $\eta_{\mathrm{tra}}$	0.945	[7,23]
Solar absorptivity $\eta_{\mathrm{abs}}$	0.94	[7,23]
Clean factor $\eta_{\mathrm{clc}}$	0.95	[7,23]
Mirror utilization rate $\eta_{\phi }$	0.91	-
Inlet/outlet temperature of HTF	315.9 °C/395.0 °C	-
Inlet/outlet pressure of HTF	5.0 MPa/3.5 MPa	-
Heat loss of pipeline	20 W/m^2^	[24]
Thermal energy storage capacity	40 L/kW∙h	[25]

**Table 3:** Model parameters of the ORC process.

Parameters	Value	Unit
Exhaust gas temperature	91	°C
Exhaust gas mass flow rate	640	kg/s
Cooling water temperature	20	°C
Isentropic efficiency of turbine	87 [13]	%
Isentropic efficiency of pump	85 [13]	%
Pump discharge pressure	2.5	MPa

**Table 4:** Thermodynamic parameters of selected organic working fluid.

Working Fluid	Boiling Point (°C)	Critical Temperature (°C)	Critical Pressure (MPa)	ODP	GWP	ASHRAE	Ref.
R113	47.59	214.06	3.39	0.9	5330	A1	[26]
R245ca	25.13	174.42	3.93	0	640	-	[26]
Rc318	−5.98	115.23	2.78	0	8700	A1	[26]
R227ea	−16.45	101.65	2.93	0	3500	A1	[26]

**Table 5:** Model parameters of the ORC components.

Main Components of ORC	Capital Cost Function (Z_CI_)	Ref.
Expander	4405 × ${\dot{W}}_{\mathrm{Exp}}^{0.89}$	[27]
Evaporator	1397 × ${\dot{A}}_{\mathrm{Evap}}^{0.89}$	[27]
Condenser	1397 × ${\dot{A}}_{\mathrm{Cond}}^{0.89}$	[27]
Pump	1120 × ${\dot{W}}_{P}^{0.8}$	[27]

**Table 6:** Main parameters selected in economic analysis.

Main Components of ORC	Capital Cost Function (Z_CI_)	Ref.
Annual operational hours, *N* (h)	7000	[29]
Annual interest, *i* (%)	14	[30]
The system life time, *n* (a)	20	[31]
Maintenance factor, φ	1.06	[31]

## Nomenclature

AC	Air Compressor
CC	Combustion Chamber
CCHP	Combined Cooling Heating and Power
CP	Condensate Preheater
DSG	Direct Steam Generation
DNI	Direct Normal Irradiance
Gen	Generator
GT	Gas Turbine
HEC	High Pressure Economizer
HEV	High Pressure Evaporator
HP	High Pressure
HPFWP	High Pressure Feed Water Pump
HRSG	Heat Recovery Steam Generator
HSH	High Pressure Superheater
HT	High Pressure Steam Turbine
HTF	Heat Transfer Fluid
IEC	Intermediate Pressure Economizer
IEV	Intermediate Pressure Evaporator
IP	Intermediate Pressure
IPFWP	Intermediate Pressure Feed Water Pump
ISCC	Integrated Solar Combined Cycle
ISH	Intermediate Pressure Superheater
IT	Intermediate Pressure Steam Turbine
LEV	Low Pressure Evaporator
LHV	Lower Heating Value
LP	Low Pressure
LPFWP	Low Pressure Feed Water Pump
LSH	Low Pressure Superheater
LT	Low Pressure Steam Turbine
NGCC	Natural Gas Combined Cycle
ORC	Organic Rankine Cycle
PTC	Parabolic Trough Collector
REH	Reheater
SRI	Solar radiation intensity
$\dot{E}x$	Exergy of a stream
$\dot{E}x_{ph}$	Physical exergy
$\dot{E}x_{ch}$	Chemical exergy
$\dot{E}x_{i}$	Exergy received by the collector
$\dot{E}x_{c}$	Exergy absorbed by the absorber
$\dot{E}x_{D,k}$	Exergy destruction of *k*th component
$\dot{E}x_{F,k}$	Fuel exergy of *k*th component
$\dot{E}x_{p,k}$	Product exergy of *k*th component
$Q_{s}$	Energy received by the collector
$Q_{a}$	Energy absorbed by the absorber
K	Incidence angle modifier
$T_{a}$	Ambient temperature
$T_{s}$	Solar surface temperature
$T_{r}$	Collectors surface temperature
$\eta_{\mathrm{int}}$	Intercept factor
$\eta_{\mathrm{ref}}$	Mirror reflectivity
$\eta_{\mathrm{tra}}$	Glass transmissivity
$\eta_{\mathrm{abs}}$	Solar absorptivity
$\eta_{\mathrm{clc}}$	Clean factor
$\eta_{\phi }$	Mirror utilization rate
$y_{D,k}$	Exergy destruction rate
